# High-Temperature Performance of Ferritic Steels in Fireside Corrosion Regimes: Temperature and Deposits

**DOI:** 10.1007/s11665-016-2423-7

**Published:** 2016-11-17

**Authors:** T. Dudziak, T. Hussain, N. J. Simms

**Affiliations:** 1 Foundry Research Institute, Centre for High Temperature Studies, Zakopiańska 73, 30-418 Kraków, Poland; 2 Faculty of Engineering, University of Nottingham, Nottingham, NG7 2RD UK; 3 Centre for Power Engineering, Cranfield University, Bedfordshire, MK43 0AL UK

**Keywords:** biomass co-firing, coal-ash corrosion, deposit induced corrosion, fireside corrosion, superheater/reheater corrosion

## Abstract

The paper reports high temperature resistance of ferritic steels in fireside corrosion regime in terms of temperature and deposits aggressiveness. Four candidate power plant steels: 15Mo3, T22, T23 and T91 were exposed under simulated air-fired combustion environment for 1000 h. The tests were conducted at 600, 650 and 700 °C according to deposit-recoat test method. Post-exposed samples were examined via dimensional metrology (the main route to quantify metal loss), and mass change data were recorded to perform the study of kinetic behavior at elevated temperatures. Microstructural investigations using ESEM-EDX were performed in order to investigate corrosion degradation and thickness of the scales. The ranking of the steels from most to the least damage was 15Mo3 > T22 > T23 > T91 in all three temperatures. The highest rate of corrosion in all temperatures occurred under the screening deposit.

## Introduction

Fireside corrosion is a major problem for fossil fuel power plants where coal is used as a fuel (Ref 1). Fireside corrosion is the metal loss of the heat exchangers due to chemical reaction with the deposits and combustion gases at high temperature. Fireside corrosion is a leading cause of tube failure in pulverized fuel power plants, and it is classified as a life-limiting factor. The tube failures due to fireside corrosion typically occur though gradual metal loss or formation of cracks. These failures are difficult to repair and can result in unplanned shutdown of the power plant resulting in significant loss of revenue. The mechanism of fireside corrosion depends on the fuel composition, firing conditions and the resultant deposit chemistry (Ref 2). Fossil fuel such as coal contains several impurities such as sulfur, chlorine, vanadium, potassium, sodium which are released when combusted in power plants (Ref 3). These corrosive species can deposit on the metal surfaces (waterwall, heat exchangers) via a number of mechanisms such as inertial impact, condensation, Brownian motion, eddy. Once deposited on the metal surface, these species can chemically react with the structural steels and fireside corrosion starts. Fireside corrosion in fossil fuel boilers are exacerbated by addition of biomass in the fuel mix (co-firing). Biomass, especially herbaceous biomass, contains higher levels of K and Cl but much less S compared to coal (Ref 3, 4). However, co-firing low levels of biomass in large power generation units is an efficient way to introduce low-carbon fuel into the electricity generation market, and the smaller dedicated biomass-fired plants tend to be less efficient (Ref 5, 6). Climate change is the major challenge to the human civilization, and it has been long established that greenhouse gases (GHG) play a major role in the global warming. According to latest International Energy Agency (IEA) energy statistics, energy activities are responsible for 69% of the anthropogenic GHG, of which 90% is CO_2_ (Ref 7). Overall, fossil fuels account for 82% of the total primary energy supply. Conventional solid fossil fuel power stations are a key contributor to this CO_2_ emission (Ref 8). Due to this fact, it is predicted that world coal demand is projected to drop by nearly 36% in 2035 (Ref 9). The world electricity demand is projected to grow annually by 2.2% between 2008 and 2035 (from approx. 16819 TW h to about 30300 TW h) (Ref 10). The new legislations will result in a decrease in subcritical power plants from 73% in 2008 (total energy market in EU) to 31% in 2035 due to development of more efficient and sustainable technologies such as ultra-supercritical (USC) power plants (Ref 9). In the UK only, 36-40% of the electricity is produced from coal-fired power stations, whereas in Poland and other east European countries, more than 80% of electricity is produced from hard coal or lignite. All these power plants operate in subcritical conditions (steam pressure around 140-160 bar and temperature 560 ^o^ C). Overall efficiencies of these plants are about 36% (up to 40%), as opposed to 45% in ultra-supercritical power plants operating at 700 °C. Each 1% increase in overall efficiency can result in as much as 3% reduction in CO_2_ emissions (Ref 11). Conventional low ferritic steels have good thermal conductivity and show acceptable fireside corrosion resistance at temperatures up to 550 °C; however, at higher temperatures such as 600 °C and above, ferritic steels with low Cr content undergo accelerated corrosion degradation due to the formation of non-protective thick scale. The effect of fireside corrosion degradation of common boiler steels is well documented in the literature (Ref 12, 13). A wide range of research in the field of coal power plants has shown that molten alkali-iron tri-sulfates can form in the deposits on heat exchangers and are very aggressive in nature (Ref 1). In a power plant, the heat exchanger materials have to survive approximately 100,000 h in operation and it is impractical to test the materials for such a long time in a laboratory-scale controlled-environment test. Therefore, two approaches are typically taken to accelerate the tests: One is to increase the test temperature to expose the materials for a shorter time period and the second is to accelerate corrosion degradation by means of a more aggressive deposit. This study presents a comprehensive investigation on the concentration of aggressive alkali-iron tri-sulfate in synthesis ash deposits at three exposure temperatures. Median metal loss of commonly used power plant steels: 15Mo3 (0.2% Cr), T22 (2.3% Cr), T23 (2.2% Cr with W, Nb) and T91 (9% Cr), under simulated air-firing combustion gases with and without various deposit conditions was studied. Dimensional metrology was used as the main tool for metal loss measurement; the scale/deposit of the corroded steels was examined using an environmental scanning electron microscopy (ESEM) with energy-dispersive x-ray analysis (EDX). The authors have previously published an article on the kinetic behavior and metal loss of 15Mo3, T22, T23 and T91 only at 650 °C with one deposit (Ref 14). A selection of the data has been reused in this comprehensive paper to describe the effect of temperature and deposit flux.

## Experimental Procedure

### Materials

One low-alloyed steel (15Mo3), two ferritic steels (T22 and T23) and one ferritic martensitic steel (T91) were used in this study (commonly known steels used in coal-fired power plants). The nominal chemical compositions of all four materials are shown in Table 1. The materials were sourced as long boiler tubes from EON Technologies (Ratcliffe) Ltd, Nottingham, UK. Each of these materials was cut and machined to a UK 600 grit surface finish (Ra < 0.4 μm). The materials were machined into tube segments, which had dimensions of ~15 mm long, ~10 mm wide (chord) with a 4-mm wall thickness.

**Table 1:** Nominal compositions of the alloys used in fireside corrosion testing (wt.%)

Alloy	Fe	C	Mn	P	S	Si	Cr	Ni	Mo	V	W	Nb	B	Al	N	Cu
15Mo3	Bal.	0.16	0.65	0.035	0.035	0.35	0.20	…	0.30	…	…	…	…	…	…	…
T22	Bal.	0.10	0.5	0.025	0.025	0.50	2.30	…	1.00	…	…	…	…	…	…	…
T23	Bal.	0.06	0.46	0.001	0.014	0.20	2.18	0.14	0.08	0.25	1.54	0.05	0.0023	0.001	0.0023	…
T91	Bal.	0.10	0.45	0.003	0.009	0.12	8.36	0.21	0.90	…	…	…	…	0.022	0.48	0.17

### Fireside Corrosion Test Setup and Exposure Conditions

**Fig. 1:**
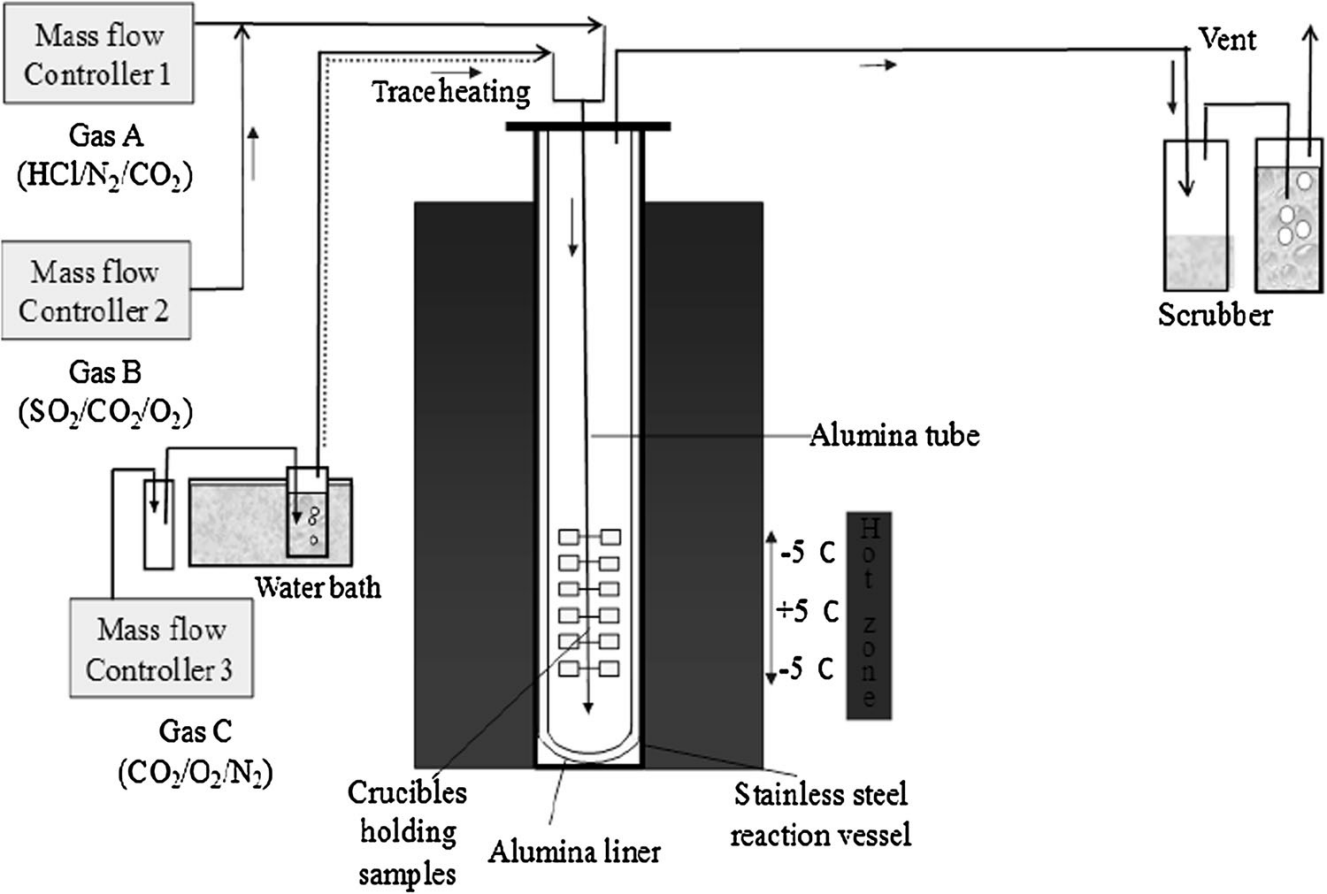
Schematic diagram of a vertical controlled-atmosphere furnace setup for fireside corrosion exposures in simulated air-fired combustion gas

Fireside corrosion tests were carried out in vertical controlled-atmosphere furnace at three exposure temperatures at 600, 650 and 700 °C for 1000 h. A schematic diagram of the furnace setup is shown in Fig. 1. The furnace is lined with high-purity alumina, and it can accommodate 24 samples in 24 individual crucibles. All the frames to hold the alumina crucibles were also made from high-purity alumina, thus avoiding any unwanted catalytic effect on the combustion gases. The exposure conditions for the tests was set following a detailed study of the gas and deposit condition that could be found around superheaters/reheaters in pulverized fuel-fired power plants using a common UK Midland’s coal (Daw Mill) with a biomass fuel (cereal co-product, CCP) available for use in the UK power stations (Ref 9). The gaseous conditions for the fireside tests were based on co-firing 80:20 wt.% of Daw Mill: CCP. The compositions of these fuels are available in previous publications (Ref 4, 6). The gas compositions produced by these fuels have been calculated using models that have been validated with pilot plant data (Ref 15, 16).

The gas compositions have been simplified to their key active components for corrosion testing in superheater/reheater environments and are shown in Table 2. Premixed gases were supplied to the controlled-atmosphere furnace through mass flow controllers to achieve the desired gas composition. The total gas flow rate during the test was ~100 cc/min. The gas containing CO_2_, O_2_ and N_2_ was passed through a de-ionized water bottle, which was kept at 40 °C in a heated water bath to add the required amount of moisture to the gas stream (8.6 vol.%) before mixing with the corrosive species (HCl and SO_2_). The flange of the furnace was kept at 40 °C to avoid any condensate build-up inside the furnace, which can result in blocking the exhaust line. The gases coming out from the furnace passed through an empty bottle to trap the condensate produced and were neutralized by passing through a scrubber of NaOH solution before being vented in the atmosphere. Each test was run for 1000 h using the well-established “deposit-recoat” technique, where every 200 h flux of deposit was achieved by adding a fresh portion of deposit (Ref 17, 18). The samples were cleaned before exposure in an ultrasonic bath in isopropanol and de-ionized water. The dimensions of each sample were measured using a digital micrometer with a resolution of ±0.001 mm. The samples with deposits were painted using a paint brush to apply a deposit loading of ~20 mg/cm^2^ on one side of the samples (outside of the tube sectioned samples). Three different deposit conditions were used in this study: the samples under the gaseous environment without any deposit, a screening D1 deposit (highly corrosive mixture to identify and eliminate steels with poor resistance, chemically) rarely observed in coal-fired power plants and a more realistic deposit D2 chemically similar to the deposit accessible in coal-fired power plants (Ref 1, 3). The chemical compositions of the deposits used in this study are shown in Table 3. These deposits have been widely used in previous studies (Ref 14, 19, 20). Deposit D1 is a standard screening deposit composition that is widely used in screening tests; it represents a composition of alkali-iron tri-sulfate that has been identified from many investigations as the principal cause of fireside corrosion in superheaters/reheaters in coal-fired power stations. Deposit D1 was diluted with kaolinite (Al_2_O_3_ 2SiO_2_ 2H_2_O) (synthetic ash) with a melting temperature of 1750 °C (Ref 21) and CaO with a melting temperature of 2572 °C (Ref 22) to represent the clay minerals usually found in coals and biomass. Diluted deposit D1 in combination of kaolinite and CaO forms deposit D2 in this work. All the deposits were mixed with isopropanol to make thick slurries for painting. The tests were cycled every 200 h and repainted with deposits to replenish the salts. The flux of deposits was 100 µg/cm^2^/h. The samples were weighted every 200 h with and without the crucibles as well as before and after applying the deposits. The measurements were taken using a digital balance with a resolution of 0.01 mg.

**Table 2:** Gas composition used in fireside corrosion tests (vol.%)

N_2_	O_2_	CO_2_	SO_2_	HCl	H_2_O
%	%	%	vppm	vppm	%
74	4	14	1298	399	8

**Table 3:** Chemical composition (mol.%) of the deposits D1 and D2 used in this study

Deposit	Fe_2_O_3_	Na_2_SO_4_	K_2_SO_4_	Al_2_O_3_.2SiO_2_.2H_2_O	CaO
No deposit	…	…	…	…	…
D1	25	37.5	37.5	…	…
D2	28	5	5	57	5

### Sample Preparation and Metal Loss Measurements

Following the 1000-h exposure, the samples were cold-mounted with glass beads according to the procedure described in detail in the other papers (Ref 6, 14, 19, 20). The polished cross sections were measured using an image analyzer to generate accurate measurements of the amount of metal remaining after the fireside corrosion tests. The dimensions of the samples were measured using a micrometer before the exposure. The measurement method has been presented in detail in previous publications by the same authors (Ref 6, 20, 23). Finally, the exposed samples were investigated using environmental scanning electron microscope ESEM (Philips XL 30) to study the scale and deposit microstructures on the polished cross sections. The microstructural characterization was conducted in backscattered electron (BSE) mode at 20 kV along with EDX analysis to identify the composition of the scale/deposits on the cross sections.

## Results

### Dimensional Metrology of the Steels

**Fig. 2:**
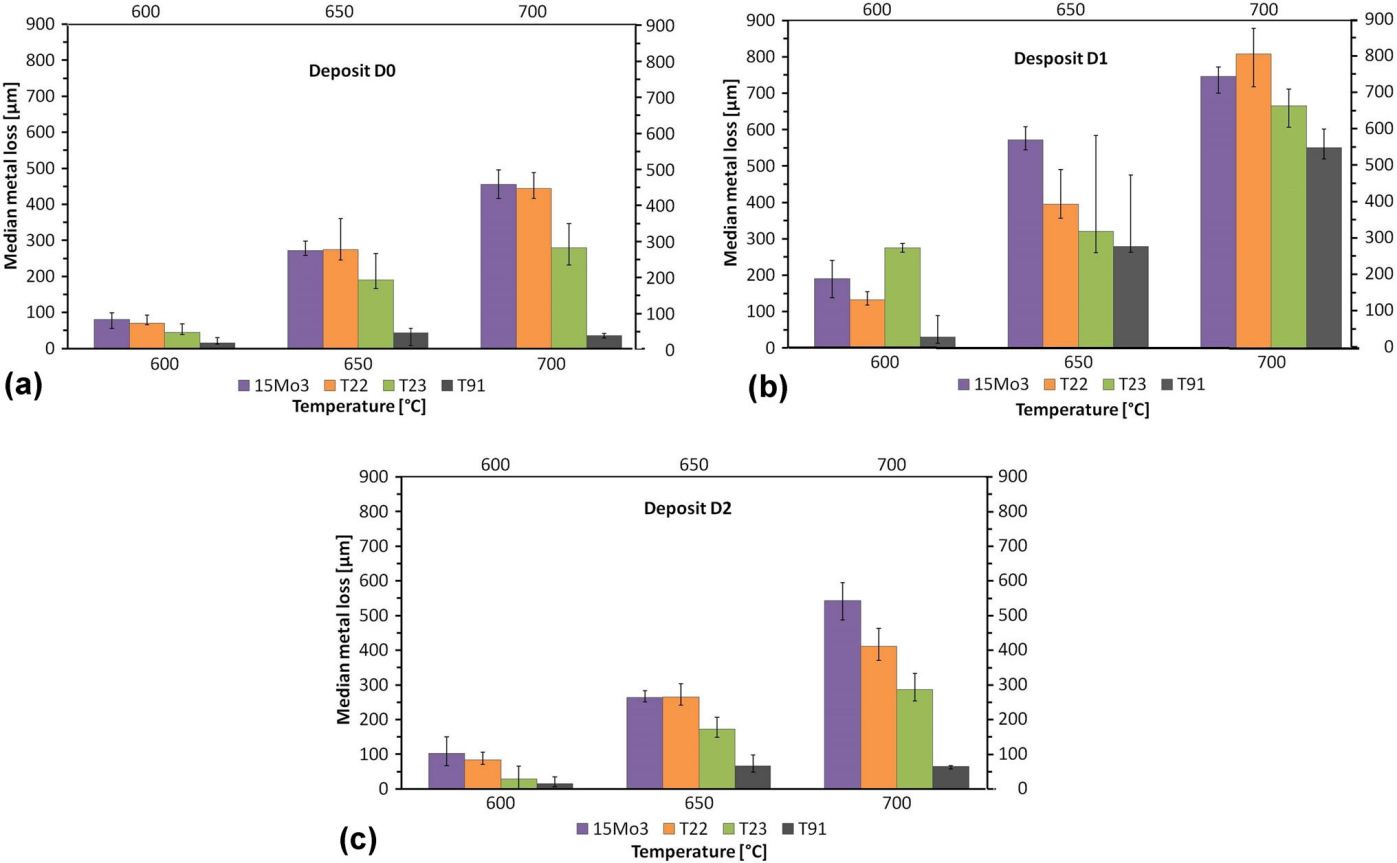
Median metal loss vs. cumulative probability plots for 15Mo3, T22, T23 and T91 at 600, 650 and 700 °C with (a) under fireside atmosphere, (b) deposit D1 and (c) deposit D2

Dimensional metrology is one of the most reliable measurement techniques available for high-temperature corrosion, as it produces a distribution of change in metal or metal loss as function of cumulative probability (Ref 18, 24, 25). Median metal loss values of 15Mo3, T22, T23 and T91 at 600, 650 and 700 °C under corrosive gases without any deposit and with deposit D1 and D2 are plotted in Fig. 2 with minimum and maximum values as error bars (the change in metal values were multiplied by -1 to present the median metal loss values as positive). Figure 2(a) shows the effect of fireside corrosion atmosphere (without deposits) on all four steels at three temperatures. In all three temperatures, the median metal loss values decreased in the following sequence 15Mo3 > T22 > T23 > T91. The 15Mo3, T22 and T23 steels show an increasing median metal loss values with increasing temperature, whereas T91 show the highest median metal loss value at 650 °C. This increasing-decreasing median metal loss trend of T91 steel was also noticed when the steel was exposed with deposit D2 (Fig. 2(c)). No such behavior of the T91 steel is observed under screening deposit D1 (Fig. 2b), which shows increase in damage for all four steels with increase in exposure temperature.

### Specific Mass Change Analysis

**Fig. 3:**
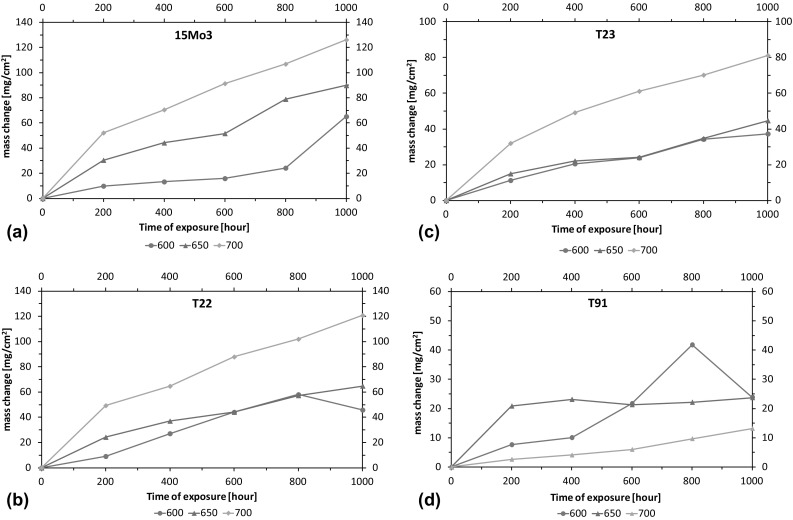
Mass change vs. exposure time for (a) 15Mo3, (b) T22, (c) T23 and (d) T91 at 600, 650 and 700 °C under fireside atmosphere for 1000 h

Traditional mass change data for all four steels at 600, 650 and 700 °C with all three deposit conditions were recorded according to the draft standards (Ref 18, 25, 26) of high-temperature corrosion, and as an example, the mass change for all four samples under fireside corrosion atmosphere without deposit is shown in Fig. 3. Mass change is the most convenient and most frequently reported method of observing metal corrosion at high temperatures; however, there are many well-known drawbacks to using mass change data, such as the spalling of oxides, and/or deposits during the course of an exposure or the formation of volatile phases (Ref 25, 27). These make it difficult to interpret the mass change data and limit its use. Nonetheless, the data for corrosion under fireside atmosphere without deposit are reported in this paper to provide a means for comparison with other published data, and the data are only shown here as reference. Figure 3(a) shows 15Mo3 steel kinetics; mass change was 60 mg/cm^2^ at 600 °C to 130 mg/cm^2^ at 700 °C, and after 1000 h, it was observed to be suppressed at 650 °C. The curves do not represent typical parabolic behavior of simple oxidation of steel. This behavior suggests accelerated corrosion of the steels due to the formation of less adherent and protective scales. Figure 3(b) shows the mass change in T22 at the three test temperatures. The final mass gain of T22 at 700 °C was quite similar to that of 15Mo3. The T22 at 600 °C suffered from mass loss after 800 h of exposure as can be seen from the decrease in the mass change. The T22 steel at 600 and 650 °C followed similar mass change behavior. In comparison with T22, the final mass change values of T23 (Fig. 3c) at all three temperatures are slightly lower. Finally, the mass change data for T91 at all three temperatures are shown in Fig. 3(d). The mass change in T91 are much smaller (less than 45 mg/cm^2^ in all three temperatures) compared to the rest of the steels; however, it appears that the steel has suffered from significant scale spallation at 600 °C after 800 h, and in other cases, mass change in T91 steel could be much higher than 80 mg/cm^2^. It is interesting to note that the mass gain of the T91 steel at the highest temperature after 1000 h was also the lowest (~12 mg/cm^2^).

### Microstructural Observations

**Fig. 4:**
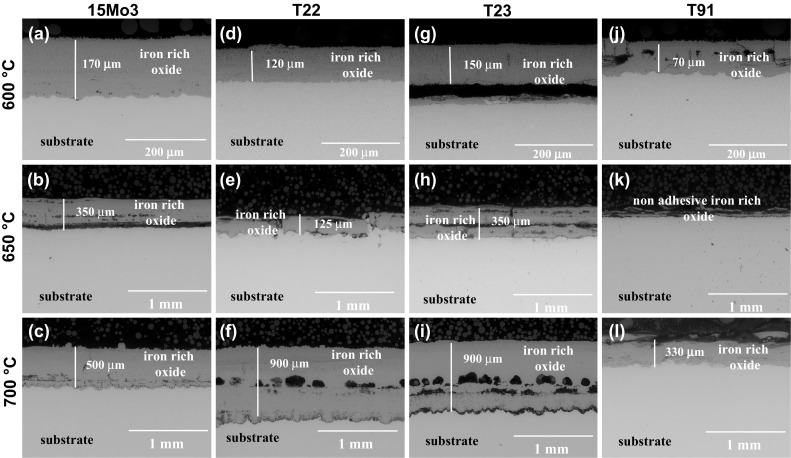
BSE images of the polished cross section of the exposed samples with deposit D0. (a, b, c) 15Mo3, (d, e, f) T22, (g, h, i) T23 and (j, k, l) T91 at 600, 650 and 700 °C for 1000 h

**Fig. 5:**
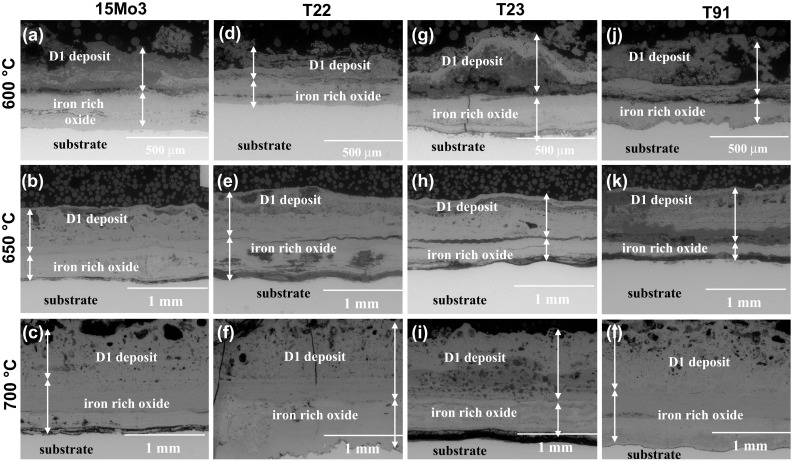
BSE images of the polished cross section of the exposed samples with deposit D1. (a, b, c): 15Mo3, (d, e, f): T22, (g, h, i): T23 and (j, k, l): T91 at 600, 650 and 700 °C for 1000 h

**Fig. 6:**
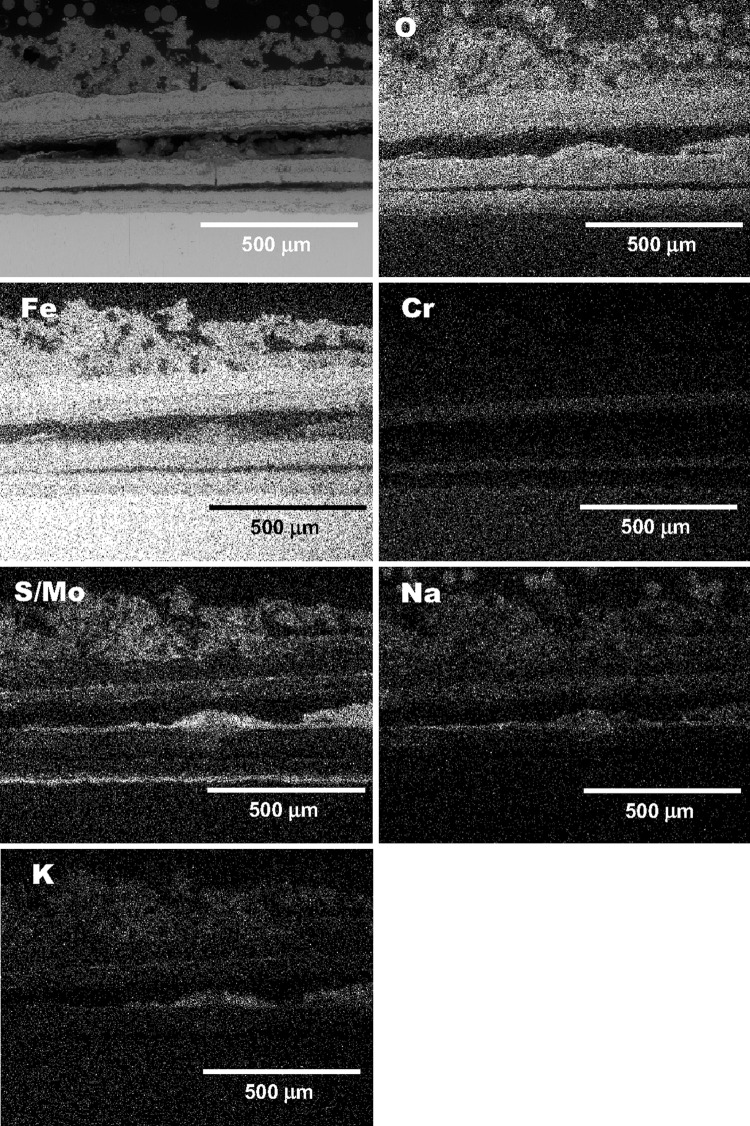
An example of EDX x-ray mapping for T22 alloy to show element distribution of the exposed samples in air-fired corrosion with D1 deposit at 600 °C

Figure 4 shows the polished cross section of the four steels after 1000-h exposure in fireside corrosion atmosphere without any deposit which were exposed at 600, 650 and 700 °C. In general, the steels showed poor fireside corrosion resistance regardless of the exposure with or without deposit. The thickness of the scale reached over 1000 µm in some samples. The voids were found in the oxide scales in T22 and T23 steels. The exposure of low-alloyed steels at all three elevated temperatures showed similar morphologies, i.e., iron-based thick oxide scales (with some variations depending of the steel chemical compositions, however, with low impact on general corrosion behavior). EDX analyses performed on the sample cross sections after 1000 h of exposure without any deposits show that the levels of Fe and O are similar at elevated temperatures between all four steels. As is shown in further part of the study on EDX mapping (Fig. 6), sulfur was detected in the oxide scales, suggesting a sulfidation attack mechanism of the four steels in this test condition. The phases constituted in 15Mo3, T22, T23 and T91, respectively, in air-fired fireside corrosion regime at 650 °C were reported earlier by Dudziak et al. (Ref 14). In this current paper, the comparison of the results at 600 and 700 °C with those from 650 °C (Ref 14) indicates that the oxide scales formed at 600 and 700 °C are similar to those 650 °C with some variations; however, the variations are not significant for overall corrosion behavior at high temperatures. Figure 5 shows the polished cross sections of 15Mo3, T22, T23 and T91 steels after exposure with deposit D1 at all three temperatures. The samples were covered in thick deposits, and XRD is not a suitable technique for these samples; hence, detailed EDX results in the form of EDX mapping shown in Fig. 6 carried out on the exposed T22 steel with D1 deposit are presented.

Figure 6 shows an example of EDS x-ray mapping performed on T22 steels exposed to show element distribution within the D1 and the oxide scale formed underneath D1 deposit. The example shown in Fig. 6 represents typical cross-sectional microstructure observed on the other alloys exposed in this work. Due to high number of samples investigated under SEM and similar degradation process found in temperature range 600-700 °C, in this work only one sample has been examined via EDS x-ray mapping.

All steels exposed in this work suffered from relatively high sulfur, sodium and potassium content. Among the three elements (sulfur, sodium and potassium), sulfur showed the highest rate of diffusion and reached scale-substrate interface. Sulfur formed an enriched band at the scale-substrate interface, whereas other elements (sodium and potassium) stayed in the D1 deposit. The highest concentration of sulfur at the interface was found in T22, T23 and T91 steels at 600 °C, and slightly lower concentration of sulfur was found at 650 °C, whereas the concentration of sulfur again increased at 700 °C. The porosity of the deposit, thickness of the scale and porosity of the oxide scale, all these influence inward diffusion of sulfur. Two distinctive areas can be noticed in the images for the samples exposed at 600 °C: the mixed scale deposit top layer and the bottom oxide layer. The top layer contains a mixture of iron oxides and heat-treated D1 deposit where sodium and potassium are present with concentration up to 2 wt.%. The thickness of mixed oxide-deposit D1 scale reached ~300 μm. The exposure at 650 °C with D1 deposit indicates acceleration in growth rate, and the thickness of the mixed deposit-oxide scale had increased to over 1000 μm, where mixture of sulfur, sodium and potassium with different concentration was found and is shown in the form of EDX mapping in Fig. 6. At the highest temperature of 700 °C, the mixed deposit-scale increased over 1500 μm in all four steels. In general, less adherence between the oxide scale and the substrate was noticed. Poor adherence of the scales is most probably due to a large mismatch in the coefficient of thermal expansion (CTE) of the oxides.

## Discussion

In this study, the high-temperature corrosion of the ferritic steels resulted in the formation of thick non-protective oxide scales at elevated temperatures. These voids in the oxide scales were only formed when the steels were exposed without any deposits. Increasing temperature resulted in increasing number of voids in the oxide scale. At lower temperatures (e.g., 600-650 °C), the number of voids was relatively low; therefore, in this discussion section, only the void formation mechanism taking place at 700 °C will be discussed. Typical size of a void in the oxide scale reached up to 50 μm (measured using ImageJ analysis tool). It is widely accepted that the voids are generated through the high rate of divergence of ionic flux between oxygen and iron during high-temperature exposure. The divergence of ionic flux may play an important role in the void formation in a growing oxide scale (Ref 28). Development of this divergence and the void formation originate from the defects developed within the crystal lattice under high temperature. It is well known that FeO (wüstite) phase possesses the highest number of defects out of all the Fe-based oxides Fe_2_O_3_ (hematite) and Fe_3_O_4_ (magnetite) (Ref 29, 30). The real phase formula instead of FeO should be denoted by Fe_1−*X*_O, the phase showing different defect ratios depending on the partial pressure of oxygen in the atmosphere.

Non-stoichiometry of FeO phase is pervasive for metal oxides, especially when the metal is not in its highest oxidation state (Ref 31). Although wüstite often is described as stoichiometric (ideal) phase with FeO formula, however, in real conditions FeO shows non-stoichiometry. The non-stoichiometry reflects the effortlessness of oxidation of Fe^2+^ to Fe^3+^ ion by replacing Fe^2+^ ion with two-thirds their number of Fe^3+^ ion. Thus, for every three “lacking” Fe^2+^ ions, the crystal contains two Fe^3+^ ions to balance the charge. The composition of a non-stoichiometric compound usually varies in a continuous manner over a narrow range (Ref 32). The formula for wüstite is written as Fe_1−*x*_O, where x donates deviation equal to 0.05.

Furthermore, based on the EDX quantitative analyses (not shown here, however, EDX mapping is provided), it was found that the external oxide scale consisted of different phase; in general, at the lowest temperature (600 °C), more Fe_3_O_4_ phase was observed, and more Fe_2_O_3_ was observed at the highest temperature (700 °C). Finally, the formation of sulfur-rich band was found in the samples covered with deposit and without deposit, exposed to air-fired atmosphere. Nevertheless, the sulfur-rich band was much clearly distinguished in the steels covered with D1 deposit. The enriched band developed, due to presence of reservoir of sulfur accumulated in deposited ash in the form of K_2_SO_4_ and Na_2_SO_4_. This finding is in good relation with melting temperature; the screening deposit D1 contained high concentration of Na_2_SO_4_ and K_2_SO_4_ salts with Fe_2_O_3_. The salts of Na_2_SO_4_ and K_2_SO_4_ melt at around 884 and 1069 °C, respectively. However, the mixture of Na_2_SO_4_ and K_2_SO_4_ forms a low-melting eutectic, which melts at 670 °C, and the melting temperature mainly depends on the ratio of both salts. Hence, high concentration of sulfur in the interface is induced by the formation of melting phase and higher diffusion rate of sulfur to the scale-substrate interface. Possible series of chemical reactions at high temperatures for alkali-iron tri-sulfates are shown below; SO_2_ from gas atmosphere (air-fired atmosphere) reacts with O_2_ and forms SO_3_ according to reaction (Ref 33). SO_3_ can form, as Fe_2_O_3_ can catalyze the reaction:

1 $$ {\text{SO}}_{{ 2 ( {\text{g)}}}} + \frac{1}{2}{\text{O}}_{{ 2 ( {\text{g)}}}} \to {\text{SO}}_{{ 3 ( {\text{g)}}}} $$

When deposit contains Fe_2_O_3_ to catalyze the oxidation of SO_2_ to SO_3_, then pyrosulfates can form according to the reactions below (Ref 34-37):

2 $$ {\text{K}}_{ 2} {\text{SO}}_{{ 4 ( {\text{s)}}}} + {\text{SO}}_{{ 3 ( {\text{g)}}}} \to {\text{K}}_{ 2} {\text{S}}_{ 2} {\text{O}}_{{ 7 ( {\text{l)}}}} $$

3 $$ {\text{Na}}_{ 2} {\text{SO}}_{{ 4 ( {\text{s)}}}} + {\text{SO}}_{{ 3 ( {\text{g)}}}} \to {\text{Na}}_{ 2} {\text{S}}_{ 2} {\text{O}}_{{ 7 ( {\text{l)}}}} $$

**Fig. 7:**
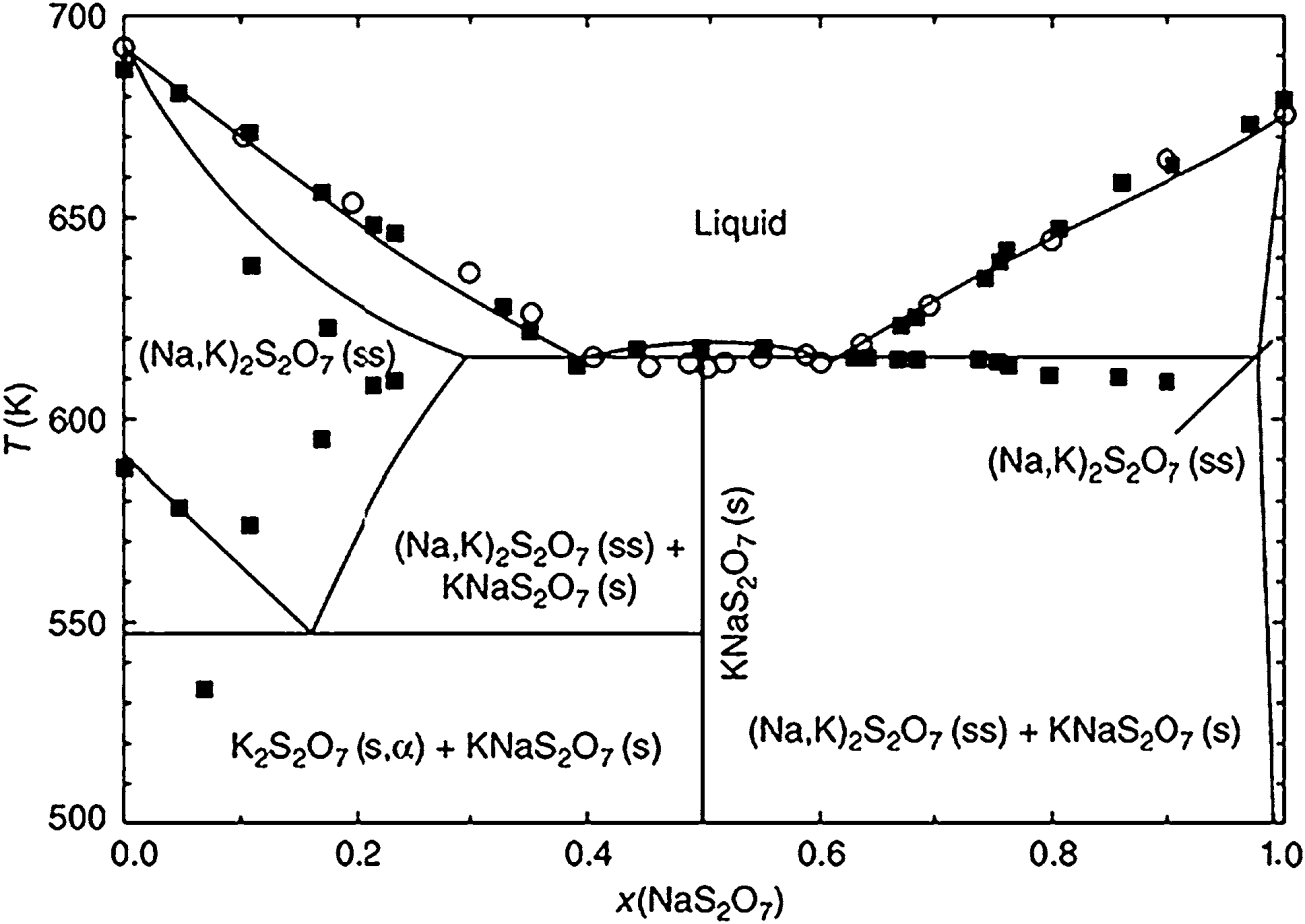
Phase diagram of alkali pyrosulfates (note temperatures in K)

Sodium and potassium pyrosulfates have much lower melting points, 398 and 454 °C, respectively, than that of pure Na_2_SO_4_ and K_2_SO_4_ individually. Coats et al. (Ref 38) examined the partial pressure of SO_3_ with the temperature of alkali pyrosulfates. Authors found that the amount of SO_3_ required to form pyrosulfates at higher temperatures (>500 °C) is higher than that needed to develop pyrosulfates at lower temperatures (<500 °C). In this study, high SO_3_ content at high temperatures (>500 °C) was associated with catalytic oxidation of SO_2_ on the exposed surfaces as showed in reaction 1. To illustrate formation of liquid phases during fireside corrosion tests, phase diagram with modeled trends has been adopted from Lindberg et al. (Ref 39) and is shown in Fig. 7. The black squares and white circles presented in Fig. 7 show experimental data from the previous studies, respectively (Ref 40). Further, the modeled phase diagram was used by other authors to predict phase development under fireside corrosion regime (Ref 41). The formation of SO_3_ accelerates the degradation by the formation of new phases with much lower melting points. Further, sodium and potassium pyrosulfates react with Fe_2_O_3_ from the oxide layer and from deposit to form alkali-iron tri-sulfates as follows:

4 $$ {\text{Me}}_{ 2} {\text{S}}_{ 2} {\text{O}}_{{ 7 ( {\text{l)}}}} + {\text{Fe}}_{ 2} {\text{O}}_{{ 3 ( {\text{s)}}}} \to 2 ( {\text{Me)}}_{ 3} ( {\text{Fe(SO}}_{ 4} )_{{ 3 ( {\text{s,l)}}}} $$

where Me denotes Na or K. It has also been reported that alkali-iron tri-sulfate phases can be formed directly without the intermediate pyrosulfate phase:

5 $$ 3 {\text{Na}}_{ 2} {\text{SO}}_{{ 4 ( {\text{s)}}}} + 3{\text{SO}}_{{ 3 ( {\text{g)}}}} + {\text{Fe}}_{ 2} {\text{O}}_{{ 3 ( {\text{s)}}}} \to 2 ( {\text{Na)}}_{ 3} {\text{Fe(SO}}_{ 4} )_{{ 3 ( {\text{s,l)}}}} + 6 {\text{O}}_{{ 2 ( {\text{g)}}}} $$

6 $$ 3 {\text{K}}_{ 2} {\text{SO}}_{{ 4 ( {\text{s)}}}} + 3{\text{SO}}_{{ 3 ( {\text{g)}}}} + {\text{Fe}}_{ 2} {\text{O}}_{{ 3 ( {\text{s)}}}} \to 2 ( {\text{K)}}_{ 3} {\text{Fe(SO}}_{ 4} )_{{ 3 ( {\text{s,l)}}}} + 6 {\text{O}}_{{ 2 ( {\text{g)}}}} $$

Both phases show relatively low melting points, sodium and potassium iron tri-sulfate melting temperature 624 and 618 °C, respectively. One of the tests was performed at 600 °C, and it can be expected that the alkali-iron tri-sulfate phases remained solid throughout whole 1000-h tests, and in two other tests, both phases should be in molten state and solidified upon cooling to room temperature; the phase iron tri-sulfate was found via XRD investigations (Ref 42). In other study, Hendry et al. (Ref 43) reported that alkali-iron tri-sulfate melts at 565 °C or even lower temperature. In the study, thermal analysis was used to determine melting temperature of alkali-iron tri-sulfate phase; the work indicates that addition of 5-30 (mol.%) Fe_2_(SO_4_)_2_ to Na_2_SO_4_-K_2_SO_4_ mixture significantly reduces melting temperature from 820 °C to below 550 °C. Thus, the formation of liquid phase in all three temperatures in this study cannot be ignored. In the case of poor scale-substrate adhesion, the substrate can react directly to the molten alkali-iron tri-sulfate through the following reaction:

7 $$ {\text{Fe}}_{{ ( {\text{s)}}}} + ( {\text{Me)}}_{ 3} {\text{Fe(SO}}_{ 4} )_{{ 3 ( {\text{l)}}}} \to {\text{Fe}}_{ 2} {\text{O}}_{{ 3 ( {\text{s)}}}} + \frac{ 3}{ 2}{\text{Fe}}_{ 3} {\text{O}}_{{ 4 ( {\text{s)}}}} + 3 {\text{Me}}_{ 2} {\text{SO}}_{{ 4 ( {\text{s)}}}} + \frac{ 3}{ 2}{\text{FeS}}_{{ ( {\text{s)}}}} $$

In air-fired combustion atmosphere, the steels can directly react with oxygen to form fresh layers of Fe_2_O_3_ and Fe_3_O_4_ depending on the partial pressure of oxygen. Thus, the newly formed oxide layer on the substrate can react with sodium and potassium pyrosulfates, and the cycle starts again. Alkali-iron tri-sulfates need to be stabilized by SO_3_ and at higher temperatures. In a previously published paper, the authors calculated the SO_2_ and SO_3_ levels over a wide range of temperatures using MTDATA for the test condition. At 600 °C, the thermodynamic equilibrium calculation shows 450 ppm SO_2_ and 850 ppm SO_3_, and at 750 °C, the levels changed to 850 ppm SO_2_ and 450 ppm SO_3_ (Ref 19). To summarize, there are two main types of sulfate reactions generally accepted under D1 conditions:

1. The formation of pyrosulfates (reactions 1-3).
2. The formation of alkali metal tri-sulfates due to the reaction with iron oxides (reactions 4-6) in contact with alkali sulfates in an oxidizing atmosphere in the presence of sulfur dioxide.

Corrosion degradation under D2 deposit showed much less impact on the low-alloyed steels at all three temperatures. The steels showed lower mass change than those under D1 deposits. Introduction of kaolinite (Al_2_O_3_ 2SiO_2_ 2H_2_O) and CaO reduced the aggressiveness of the deposit. Kaolinite acts by binding alkali phases in ash and induces the formation of potassium or sodium alumina silicates with much higher melting point than pure potassium or sodium silicates (Ref 44). Calcium-based additives, CaO added to D2, similar to kaolinite, influence the high-temperature performance of the low-alloyed steels. Development of high-melting silicates composed of calcium and alkali (Ref 45, 46) is the main factor which induces slightly better corrosion resistance of the steels under D2 deposit used in this work. Furthermore, CaO reacts with HCl and SO_2_ from the flue gas; hence, sulfur partial pressure is reduced, resulting in lower diffusion of sulfur throughout the oxide scale. In contrast, high rate of sulfur diffusion was observed under the D1 deposit where CaO was not added.

## Conclusions

This paper reports the results of a comprehensive investigation on the effect of temperature on metal loss of commonly used power plant steels: 15Mo3 (0.2% Cr), T22 (2.3% Cr), T23 (2.2% Cr with W, Nb) and T91 (9% Cr), in simulated coal-biomass combustion environment at 600-700 °C. The tests were conducted according to the deposit-recoat test method for 1000 h. Dimensional metrology was used as the major tool for quantifying the damage induced by aggressive alkali-iron tri-sulfate phases to the steels. Based on the results, the following conclusions can be drawn from this study:

1. In simulated combustion gases without any deposit, the ranking of the steels from most to the least damage was 15Mo3 > T22 > T23 > T91 in all three temperatures. The metal loss of the materials increased with increasing temperature for 15Mo3, T22 and T23, whereas T91 showed a decrease in metal loss at 700 °C due to formation of a more protective scales than that observed in other steels.
2. The oxide scales on 15Mo3, T22 and T23 (without any deposit) developed to several hundreds of micrometer in thickness, and the scales were non-protective, developed cracks, delamination and voids within the scale. The oxide scales on T91 were much thinner. Sulfur was detected in the scale, suggesting a sulfidation attack of the steels in the test temperatures.
3. All four steels covered in screening deposit (D1) suffered from high metal loss (two times compared to samples without deposits) and the median metal loss values increased with increasing temperature. The top layer of the sample was covered with mixed corrosion products/deposit, and underneath, a complex oxide scale developed due to the molten alkali-iron tri-sulfates formation. The oxide scale showed poor adherence to the substrate, especially at 650 °C. In general, the ranking of the steels from the most to the least damage was 15Mo3 > T22 > T23 > T91, with the exception at 600 °C where T23 outperformed T22.
4. Addition of CaO and Al_2_O_3_ 2SiO_2_ 2H_2_O phases to the D2 deposit at high-temperature air-fired atmosphere developed phases with high melting points; hence, lower corrosion degradation of the low-alloyed steels was observed.

## References

[1] Stringer J, Wright IG (1995). Current Limitations of High-Temperature Alloys in Practical Applications. Oxid. Met..

[2] Cutler AJB, Raask E (1981). External Corrosion in Coal-Fired Boilers: Assessment From Laboratory Data. Corros. Sci..

[3] Nielsen HP, Baxter LL, Sclippab G, Morey C, Frandsen FJ, Dam-Johansen K (2000). Deposition of Potassium Salts on Heat Transfer Surfaces in Straw-Fired Boilers: A Pilot-Scale Study. Fuel.

[4] Simms NJ, Sumner J, Hussain T, Oakey JE (2013). Fireside Issues in Advanced Power Generation Systems. J. Mater. Sci. Technol..

[5] Aho M, Silvennoinen J (2004). Preventing Chlorine Deposition on Heat Transfer Surfaces with Alumnium-Silicon Rich Biomass Residue and Additive. Fuel.

[6] Syed AU, Simms NJ, Oakey JE (2012). Fireside Corrosion of Superheaters: Effects of Air and Oxy-Firing of Coal and Biomass. Fuel.

[7] International Energy Agency (IEA), *CO*_*2*_*Emissions from Fuel Combustion, Highlights*, IEA Publications, Paris Cedex 15, France, 2014, ISBN:978-92-64-20804-9

[8] Skea J, Ekins P, Winskel M (2011). Energy 2050: Making the Transition to a Secure Low Carbon Energy System.

[9] International Energy Agency (IEA), *World Energy Outlook*, IEA Publications, Paris Cedex 15, France, 2010, ISBN:978-92-64-08624-1

[10] Kjaer S (1996). Status and Future of Advanced pf Power-Plants. Energy Convers. Manag..

[11] Henry J, Zhou G, Ward T (2007). Lessons from the Past: Materials-Related Issues in an Ultra Supercritical Boiler at Edddystone Plant. Mater. High Temp..

[12] W. Reid, *External Corrosion and Deposits: Boilers and Gas Turbines*, Fuel and Energy Science Series, J. Beér, Ed., American Elsevier Publishing Company, Inc, New York, 1971

[13] K. Natesan, A. Purohit, D.L. Rink, Coal-Ash *Corrosion of Alloys for Combustion Power Plants*. US department of Energy Fossil Energy 17th Annual Conference on Fossil Energy Materials (2003)

[14] Dudziak T, Hussain T, Orlicka D, Pokrywa A, Simms N (2015). Fireside Corrosion Degradation of 15Mo3, T22, T23 and T91 in Simulated Coal-Biomass Co-fired Environment. Mater. Corros..

[15] Simms NJ, Fry AT, Lecomte-Beckers J, Carton M (2010). Modelling Fireside Corrosion of Heat Exchangers in Co-fired Pulverised Fuel Power Systems. Materials for Advanced Power Engineering.

[16] Simms NJ, Kilgallon PJ, Oakey JE (2007). Fireside Issues in Advanced Power Generation Systems. Energy Mater. Mater. Sci. Eng. Energy Syst..

[17] Saunders RJ, Grabke HJ, Meadowcroft DB (1995). Guidelines for Methods of Testing and Research in High Temperature Corrosion.

[18] Draft Code of Practice: TESTCORR—Discontinuous Corrosion Testing in High Temperature Environment ERA Report 2000-0546, Surrey, The United Kingdom (2000)

[19] Hussain T, Syed AU, Simms NJ (2013). Trends in Fireside Corrosion Damage to Superheaters in Air and Oxy-Firing of Coal/Biomass. Fuel.

[20] Dudziak T, Hussain T, Simms NJ, Syed AU, Oakey JE (2014). Fireside Corrosion Degradation of Ferritic Alloys at 600 °C in Oxy-fired Conditions. Corros. Sci..

[21] Huertas FJ, Fiore S, Huertas F, Linares J (1999). Experimental Study of the Hydrothermal Formation of Kaolinite. Chem. Geol..

[22] Kouzu M, Kasuno T, Tajika M, Sugimoto Y, Yamanaka S, Hidaka J (2008). Calcium Oxide as a Solid Base Catalyst for Transesterification of Soybean Oil and its Application to Biodiesel Production. Fuel.

[23] Dudziak T, Grobauer S, Simms N, Krupp U, Łukaszewicz M (2014). Metal Loss of Steam-Oxidized Alloys After Exposures at 675°C and 725°C for 500 Hours. High Temp. Mater. Process. (London).

[24] J.R. Nicholls, P. Hancock, Analysis of Oxidation and Hot Corrosion Data—A Statistical Approach, ed. by. A. Rapp Robert, NACE Conference, San Diego, California, USA (1983)

[25] Corrosion of Metals and Alloys-Methods for Metallographic Examination of Samples After Exposure to High Temperature Corrosive Environments, *Draft ISO Standard*. ISO/TC 156 NWI 5092005 (2006)

[26] Saunders SRJ, Grabke HJ, Meadowcroft DB (1995). Guidelines for Methods of Testing and Research in High Temperature Corrosion.

[27] Young D (2008). High Temperature Oxidation and Corrosion of Metals.

[28] Maruyama T, Ueda M, Kawamura K (2009). Void Formation in the Growing Scale Induced by the Divergence of the Diffusive Ionic Flux in High Temperature Oxidation of Metal. Defect Diffus. Forum.

[29] Hazen RM, Jeanloz R (1984). Wüstite (Fe_1-x_ O) A Review of its Defect Structure and Physical Properties. Rev. Geophys..

[30] Kofstad P, Zeev A (1968). Hed, Defect Structure Model for Wustite. J. Electrochem. Soc..

[31] Greenwood NN, Earnshaw A (2012). Chemistry of the Elements.

[32] Lesley Smart E (2012). Solid State Chemistry: An Introduction.

[33] Tomeczek J (2007). Corrosion Modelling of Austenitic Steel in Molten Sulphate Deposit. Corros. Sci..

[34] Ståhl K, Balic-Zunic T, da Silva F, Eriksen KM, Berg RW, Fehrmann R (2005). J. Solid State Chem..

[35] Karlsson A, Moller P, Johansen V (1990). Iron and Steel Corrosion in a System of O_2_, SO_2_ and Alkali Chloride. The Formation of Low Melting Point Salt Mixtures. Corros. Sci..

[36] Harb JN, Smith EE (1990). Fireside Corrosion in PC-Fired Boilers. Prog. Energy Combust..

[37] P. Rademakers, W. Hesseling, J van de Wetering, Review on Corrosion in Waste Incinerators, and Possible Effects of Bromine. TNO Report (2002)

[38] Coats AW, Dear DJA, Penfold D (1968). Phase Studies on the Systems Na_2_SO_4_–SO_3_, K_2_SO_4_–SO_3_, and Na_2_SO_4_–K_2_SO_4_–SO_3_. J. Inst. Fuel.

[39] Lindberg D, Backman R, Chartrand P (2001). Thermodynamic Evaluation and Optimization of the (NaCl + Na_2_SO_4_ + Na_2_CO_3_ + KCl + K_2_SO_4_ + K_2_CO_3_) system. J. Chem. Thermodyn..

[40] Rasmussen SB, Eriksen KM, Hatem G, Da Silva F, Staahl K, Fehrmann R (2001). Conductivity, Thermal Measurements, X-ray Investigations, and Phase Diagram of the Na_2_S_2_O_7_-K_2_S_2_O_7_ system. J. Phys. Chem. B.

[41] N.J. Simms, *Power Plant Life Management and Performance Improvement*, J. Oakey, Ed., Woodhead Publishing Limited, Cambridge, 2011

[42] A. Syed, Fireside Corrosion Study of Superheater Materials in Advanced Power Plants, PhD Thesis, Cranfield University (2011)

[43] Hendry A, Lees DJ (1980). Corrosion of Austenitic Steels in Molten Sulphate Deposits. Corros. Sci..

[44] Carroll JP, Finnan JM (2015). The Use of Additives and Fuel Blending to Reduce Emissions from the Combustion of Agricultural Fuels in Small Scale Boilers. Biosyst. Eng..

[45] Wang L, Hustad JE, Skreiberg Ø, Skjevrak G, Grønli M (2012). A Critical Review on Additives to Reduce Ash Related Operation Problems in Biomass Combustion Applications. Energy Procedia.

[46] Bäfver LS, Rönnbäck M, Leckner B, Claesson F, Tullin C (2009). Particle Emission from Combustion of Oat Grain and its Potential Reduction by Addition of Limestone or Kaolin. Fuel Process. Technol..

